# Acceptability of self-collected vaginal swabs for sexually transmitted infection testing among youth in a community-based setting in Zimbabwe

**DOI:** 10.1177/09564624231152804

**Published:** 2023-03-24

**Authors:** Helena Rochford, Leyla Larsson, Victoria Simms, Constancia Mavodza, Lovemore Sigwadhi, Ethel Dauya, Anna Machiha, Mitchell Mavhura, Tatenda Bipiti, Chido Mangena, Tsitsi Bandason, Rashida A Ferrand, Suzanna C Francis, Chido Dziva Chikwari

**Affiliations:** 1 The Health Research Unit Zimbabwe, 559150 Biomedical Research and Training Institute, Harare, Zimbabwe; 2 MRC International Statistics and Epidemiology Group, Department of Infectious Disease Epidemiology, 4906 London School of Hygiene & Tropical Medicine, London, UK; 3 Department of Public Health, Environments and Society, 4906 London School of Hygiene & Tropical Medicine, London, UK; 4 AIDS and TB Unit, 108271 Ministry of Health and Child Care, Harare, Zimbabwe; 5 Clinical Research Department, 4906 London School of Hygiene & Tropical Medicine, London, UK

**Keywords:** Africa, diagnosis, screening, trichomoniasis (Trichomonas vaginalis), women

## Abstract

**Background:**

Youth are a high-risk group for sexually transmitted infections (STIs). To increase access to STI testing, convenient approaches for sampling and testing are needed. We assessed the acceptability of self-collected vaginal swabs (SCVS) for STI testing among young women (16–24 years) attending a community-based sexual and reproductive health service in Zimbabwe.

**Methods:**

A SCVS was used for point-of-care testing for *Trichomonas vaginalis* and a urine sample for testing for *Chlamydia trachomatis* and *Neisseria gonorrhoeae*. A questionnaire was administered to investigate the acceptability of SCVS versus self-collected urine samples. In-depth interviews (IDIs) explored the experience of SCVS and reasons for sample collection preference. Qualitative analysis was predominantly deductive.

**Results:**

We recruited 129 women who took up STI testing (median age 20 years, IQR 18–22 years) and conducted 12 IDIs. Most participants reported that they understood the instructions (93.0%) and found SCVS easy (90.7%). Many participants felt relaxed (93.0%), in control (88.4%) and that they had enough privacy (90.7%). Pain or discomfort were reported by 16.3% and embarrassment by 15.5%. Among the 92 (71.3%) participants who provided both a SCVS and urine sample, 60.9% preferred SCVS. Sample collection method preferences were similar between 16–19 and 20–24year-olds. In IDIs, clear instructions, privacy, trust in the service and same-day results were perceived as important facilitators to taking up SCVS. Participants frequently described feeling relaxed and confident whilst taking a SCVS. Pain and discomfort were uncommon experiences.

**Conclusions:**

SCVS for STI testing are acceptable to young women and a feasible method of sample collection in community-based settings.

## Introduction

Globally, the control of sexually transmitted infections (STIs) has been suboptimal with a persistently high incidence of STIs. In 2020 the World Health Organization (WHO) estimated 96 million curable STIs cases in the African Region.^1^

Youth are a high-risk group for STI acquisition and face unique barriers to accessing STI services, including cost, stigma and judgmental attitudes of providers, confidentiality, opportunity cost and low STI literacy.^2–5^ To increase access to STI testing, flexible, acceptable, and convenient approaches for sampling and testing are needed. Testing in community-based settings and self-collected vaginal swabs (SCVS) may reduce the embarrassment associated with the provision of a sample by youth.^6^ However, acceptability data among youth and in community-based settings are limited. In addition, few studies have been conducted in low- and middle-income countries.

We assessed the acceptability of SCVS for STI testing among youth aged 16–24 years in a community-based setting in Zimbabwe.

## Methods

### Study design

This mixed methods study was nested within the CHIEDZA trial, a cluster randomised trial of community-based, integrated HIV and sexual and reproductive health services for youth aged 16–24 years in Zimbabwe (NCT03719521).^7^ The intervention included the provision of HIV testing and treatment, menstrual health information and products, contraception, counselling and STI testing. Urine was collected for *Chlamydia trachomatis* (CT) and *Neisseria gonorrhoeae* (NG) testing using GeneXpert (Cepheid, Sunnyvale, CA, USA), with results available within a week. Point-of-care testing for *Trichomonas vaginalis* (TV) using the OSOM Trichomonas Rapid Test (Sekisui, Burlington, MA, USA) and a SCVS was also offered with results available in 15 minutes. TV testing was conducted on-site according to the package inserts provided by the manufacturer. Package inserts were also shown to participants to aid sample collection. CT and NG testing was conducted in a laboratory. Services were delivered in community centres and samples were collected in toilets.

This study was conducted between June and August 2021 in four intervention communities in Harare. Youth who took up TV testing were recruited*.* Written informed consent was obtained from all participants.

A questionnaire to assess the acceptability of SCVS was developed based on a systematic review by Paudyal et al.^8^ Questions explored experiences of SCVS using a four-point Likert scale. Participants who also provided a urine sample were asked for their sampling method preference.

The sample size was calculated to compare differences in the acceptability of SCVS by age group (16–19 vs. 20–24 years). A sample size of 64 per group (128 in total) was sufficient to detect a difference in mean scores from questionnaire responses in the two groups of half a standard deviation i.e., an effect size of 0.5, with 80% power. For in-depth interviews (IDIs), a sample size of 12 was deemed to be adequate in order to explore a range of experiences of 16–19 and 20–24-year-olds.

Purposive sampling was used to select participants who had provided a SCVS for IDIs. Participants were selected for IDIs based on their questionnaire responses to ensure a variety of experiences were captured. In addition, participants from both age groups were selected. History of sexual debut was not taken into account when selecting participants for IDIs. If a participant did not attend their appointment, an alternative participant attending CHIEDZA on the day was recruited. IDIs explored the experience of obtaining a SCVS and sampling method preference.

IDIs were carried out by youth researchers in Shona or English. Youth researchers were 18–24 years old and attended a Youth Researchers Academy where they received training on research methods and were mentored to develop the study protocol and data collection tools, collect and analyse data.^9^

### Data management and analysis

Quantitative data was collected on electronic tablets using SurveyCTO (Dobility Inc, Washington, USA), transferred to a Microsoft SQL Server database for cleaning then exported to Stata v17.0 for analysis. The four-point Likert scores were recoded into binary variables. *p* values were calculated to compare the experiences of 16–19 and 20–24-year-olds using Fisher’s exact test for variables with values less than or equal to five, and Chi-squared statistic for variables with values greater than five.

IDIs were recorded, transcribed, translated, and analysed thematically. Qualitative data analysis was predominantly deductive, based on predetermined themes and findings in the quantitative data.

## Results

Overall, 129 participants were enrolled (median age 20 (IQR 18–22) years). Many participants reported having had sex 83 (64.3%), with a higher proportion of 20–24 than 16–19-year-olds (80.0% vs. 45.8%, *p* < 0.001) reporting sexual debut. No participants used tampons for menstrual management and only two used a menstrual cup. Only 37 (28.7%) participants had previously taken a SCVS (Table 1).

**Table 1.** Characteristics and experiences of self-collected vaginal swabs for young women in a community-based setting in Zimbabwe.

	Response	16–19 years	20–24 years	Total	Chi-Square	*p*
N		59 (45.7%)	70 (54.3%)	129		
Menstrual management product used at last period	Disposable pads	46 (78.0%)	46 (65.7%)	92 (71.3%)	2.35	0.13
	Reusable pads	12 (20.3%)	17 (24.3%)	29 (22.5%)	0.29	0.59
	Cotton wool	2 (3.4%)	8 (11.4%)	10 (7.8%)		0.11*
	Toilet paper	1 (1.7%)	1 (1.4%)	2 (1.6%)		1.00*
	Menstrual cup	1 (1.7%)	1 (1.4%)	2 (1.6%)		1.00*
	Tampon	0 (0.0%)	0 (0.0%)	0 (0.0%)		
Ever had sexual intercourse?	Yes	27 (45.8%)	56 (80.0%)	83 (64.3%)	16.36	<0.001
Ever self-collected a vaginal swab?	Yes	15 (25.4%)	22 (31.4%)	37 (28.7%)	0.56	0.45
How easy or difficult did you find the instructions for collecting vaginal swabs?	Very easy/easy	51 (86.4%)	69 (98.6%)	120 (93.0%)	7.26	0.01
How easy or difficult was it to self-collect the vaginal swab?	Very easy/easy	51 (86.4%)	66 (94.3%)	117 (90.7%)	2.34	0.13
Whilst self-collecting a vaginal swab						
I felt that I was provided with enough privacy	Strongly agree/agree	51 (86.4%)	66 (94.3%)	117 (90.7%)	2.34	0.13
I felt relaxed	Strongly agree/agree	56 (94.9%)	64 (91.4%)	120 (93.0%)	0.60	0.44
I felt in control	Strongly agree/agree	50 (84.8%)	64 (91.4%)	114 (88.4%)	1.39	0.24
I trusted that I was collecting the swab correctly	Strongly agree/agree	51 (86.4%)	62 (88.6%)	113 (87.6%)	0.13	0.72
I was anxious	Strongly agree/agree	29 (49.2%)	22 (31.4%)	51 (39.5%)	4.21	0.04
I felt embarrassed	Strongly agree/agree	12 (20.3%)	8 (11.4%)	20 (15.5%)	1.94	0.16
I experienced pain or discomfort	Strongly agree/agree	12 (20.3%)	9 (12.9%)	21 (16.3%)	1.32	0.25
I worried that I might not doing the test correctly	Strongly agree/agree	14 (23.7%)	19 (27.1%)	33 (25.6%)	0.20	0.66
Would you be willing to take a self-collected vaginal swab in the future?	Yes	58 (98.3%)	67 (95.7%)	125 (96.9%)	0.72	0.40
Would you recommend or encourage a friend to self-collect a vaginal swab?	Yes	59 (100.0%)	69 (98.6%)	128 (99.2%)	0.85	0.36

*p values calculated using Fisher’s exact test.

### Acceptability of SCVS

Most participants reported that they felt relaxed 120/129 (93.0%), in control 114/129 (88.4%), that they were provided with enough privacy 117/129 (90.7%) and trusted that they were self-collecting the vaginal swab correctly 113/129 (87.6%). Experiences of embarrassment, pain and discomfort were uncommon. More 20–24 than 16–19-year-olds found the instructions for SCVS easy to understand (98.6% vs. 86.4%, *p* = 0.01). Fewer participants aged 20–24 than 16–19 years reported anxiety (31.4% vs. 49.2%, *p* = 0.04) whilst self-collecting vaginal swabs. Most participants said that they would do a SCVS in the future and would recommend SCVS to a friend (Table 1).

### Importance of privacy for self-collecting samples for STI testing

IDIs showed that provision of a private space and clear instructions facilitated SCVS collection. Most participants reported positive experiences of privacy, which could be compromised by a lack of locks on toilets and/or being disturbed by their peers whilst obtaining samples (Table 2).

**Table 2.** Themes and quotes from in-depth interviews exploring the acceptability of self-collected vaginal swabs among youth in a community-based setting in Zimbabwe.

Importance of privacy for self-collecting samples for sexually transmitted infection testing
*“Privacy was okay since I was alone and there was no one to disturb me whilst sampling”* (16–19 years)
Competence in, and ease of, self-collecting vaginal swabs
*“I felt I was in control, because I had understood the instructions”* (16–19 years)
*“What made me feel relaxed is that I knew that I was doing something good for my health”* (20–24 years)
*“I was amazed that I was going to insert that item into my vagina, but I then used it and noticed that it’s not harmful”* (20–24 years)
Control, privacy and no one else touching their body
*“What helped is that I’m not used to doing such things around people so the privacy I see it as a good thing as I sampled alone”* (20–24 years)
*“It’s a good thing because you will be sampling on your own. If it was said that someone has to do it on me then yeah that would get me anxious and embarrassed but doing it on my own, you will have a lot of confidence plus all the fear will be lost”* (20–24 years)
Ease of use and lack of pain, associated with having clear instructions
*“It helped because I never experienced pain because I was told how to do it”* (16–19 years)
Trust in providers, services, and results at CHIEDZA
*“I can trust the results very well because I trust the people who were delivering the services”* (16–19 years)

### Competence in, and ease of, self-collecting vaginal swabs

Youth described how clear instructions gave them confidence and reduced their fear of SCVS. Further, taking a sample was perceived as good for their health, which facilitated feeling relaxed whilst self-sampling. Participants were positively surprised by the lack of pain and ease of use. Few were concerned about their SCVS technique (Table 2).

### Sample collection method preference

Of the 129 participants, 92 (71.3%) also provided a urine sample for CT and NG testing. Of these, 56/92 (60.9%) preferred the SCVS. Preferences were similar for 16–19 and 20–24-year-olds. Of those who had done a SCVS in the past, 16/28 (57.1%) preferred SCVS. Of the 58 participants who had previously had sexual intercourse, 36 (62.1%) preferred SCVS.

**Figure 1.**
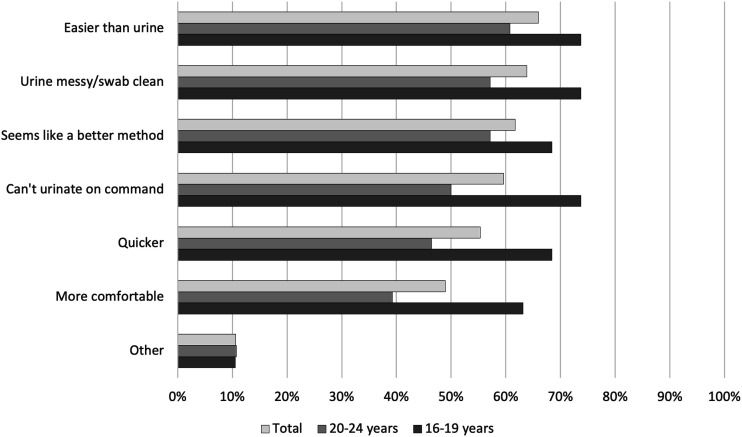
Reasons that young women prefer self-collected vaginal swabs compared to self-collected urine samples in a community-based setting in Zimbabwe.

**Figure 2.**
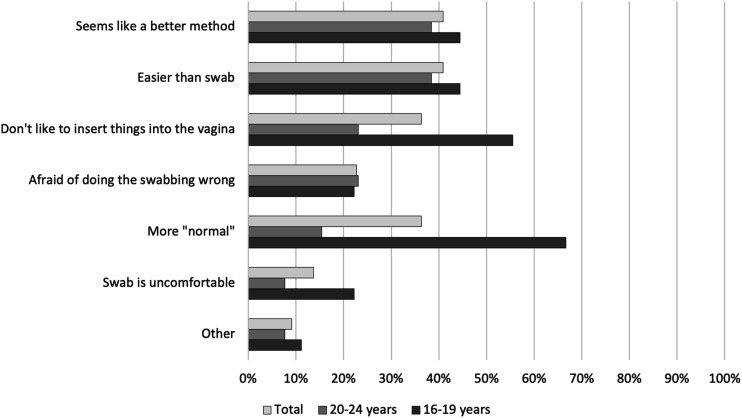
Reasons that young women prefer self-collected urine samples compared to self-collected vaginal swabs in a community-based setting in Zimbabwe.

Among those who provided a reason for preferring SCVS, 16–19-year-olds were more likely than 20–24-year-olds to report that they could not urinate on command (73.7% vs. 50.0%) and urine samples were messy and swab samples were clean (73.7% vs. 57.1%) (Figure 1). Adolescents who preferred urine samples more commonly stated reasons such as “More normal” (66.7% vs. 15.4%) and “Don’t like to insert things into the vagina” (55.6% vs. 23.1%) (Figure 2).

## A preference for SCVS

### Control, privacy and no one else touching their body

Youth said they were in control when self-collecting vaginal swabs. They were able to stop if they experienced pain rather than relying on alerting a service provider to remove the swab. Youth described feeling more comfortable self-collecting partly due to the sampling process being unfamiliar. Some did not feel embarrassed whilst self-sampling as it was them touching their body rather than someone else (Table 2).

### Ease of use and lack of pain, associated with having clear instructions

Most interviewees reported no pain or discomfort whilst obtaining SCVS. Lack of pain was attributed to self-collection being easy and receiving clear instructions (Table 2).

### Trust in providers, services, and results at CHIEDZA

Many described trust in the results from SCVS. Some reported having confidence in the results because they trusted the CHIEDZA service providers, trusted the CHIEDZA interventions and visualised the testing process (Table 2).

## Discussion

Our study showed high acceptability of SCVS for young women in a community-based setting in Zimbabwe. Most youth preferred SCVS compared to urine samples for STI testing. SCVS were acceptable to both 16–19 and 20–24-year-olds. SCVS gave the young women a sense of control and autonomy. Interestingly, SCVS were highly acceptable despite insertion methods of menstrual management such as tampons and menstrual cups being uncommon in this population.

Acceptability of SCVS has been reported in multiple settings.^10–13^ However, there are few data on the acceptability of SCVS among youth in community-based settings in Africa. One study conducted in the USA described how participants aged between 12–21 years trusted results from SCVS less than pelvic examinations by clinicians and self-collected urine samples.^14^ Youth reported concern about incorrect technique for SCVS.^14^ Youth in our study were comfortable with SCVS and trusted the results.

Younger women tended to dislike aspects of sampling methods associated with penetration resulting in some participants preferring a non-penetrative method of sample collection. Acceptability of penetrative sampling methods increased with age, which may be a result of having experienced sexual debut or more knowledge about one’s body. A choice of penetrative and non-penetrative sampling methods may be more appropriate for younger adolescents.

CHIEDZA is a community-based, youth-friendly service; clients trusted the service and the providers which may have added to their trust in SCVS. The delivery of health interventions in community-based settings is a potential alternative approach for youth to circumvent perceived barriers to accessing health facilities.

Youth-led research in our study might have facilitated the discussion of sensitive subjects and provided an “insider perspective”, making the findings more relevant.^15^ Qualitative data was collected in a short time frame, by newly trained youth researchers which affected the quality of IDIs. Participants were selected for IDIs using a combination of purposive and convenience sampling which could have resulted in selection bias. We note that test results from the SCVS were available on the same day which may have contributed to its increased acceptability.

In conclusion, this study demonstrates that SCVS are acceptable to youth in a community-based setting in Zimbabwe. SCVS should be used by researchers and service providers when providing STI testing for youth.
